# Changes in Plasma Levels of N-Arachidonoyl Ethanolamine and N-Palmitoylethanolamine following Bariatric Surgery in Morbidly Obese Females with Impaired Glucose Homeostasis

**DOI:** 10.1155/2015/680867

**Published:** 2015-03-22

**Authors:** Akhila Mallipedhi, Sarah L. Prior, Gareth Dunseath, Richard M. Bracken, Jonathan Barry, Scott Caplin, Nia Eyre, James Morgan, John N. Baxter, Saoirse E. O'Sullivan, Sarir Sarmad, David A. Barrett, Stephen C. Bain, Steve D. Luzio, Jeffrey W. Stephens

**Affiliations:** ^1^Diabetes Research Group, Institute of Life Sciences, Swansea University, Swansea SA2 8PP, UK; ^2^Department of Diabetes & Endocrinology, Morriston Hospital, ABM University Health Board, Swansea SA6 6NL, UK; ^3^Welsh Institute of Metabolic and Obesity Surgery, Morriston Hospital, ABM University Health Board, Swansea SA6 6NL, UK; ^4^Division of Medical Sciences and Graduate Entry Medicine, University of Nottingham, Royal Derby Hospital, Derby DE22 3DT, UK; ^5^Centre for Analytical Bioscience, School of Pharmacy, University of Nottingham, Nottingham NG7 2RD, UK

## Abstract

*Aim*. We examined endocannabinoids (ECs) in relation to bariatric surgery and the association between plasma ECs and markers of insulin resistance.* Methods*. A study of 20 participants undergoing bariatric surgery. Fasting and 2-hour plasma glucose, lipids, insulin, and C-peptide were recorded preoperatively and 6 months postoperatively with plasma ECs (AEA, 2-AG) and endocannabinoid-related lipids (PEA, OEA).* Results*. Gender-specific analysis showed differences in AEA, OEA, and PEA preoperatively with reductions in AEA and PEA in females postoperatively. Preoperatively, AEA was correlated with 2-hour glucose (*r* = 0.55, *P* = 0.01), HOMA-IR (*r* = 0.61, *P* = 0.009), and HOMA %S (*r* = −0.71, *P* = 0.002). OEA was correlated with weight (*r* = 0.49, *P* = 0.03), waist circumference (*r* = 0.52, *P* = 0.02), fasting insulin (*r* = 0.49, *P* = 0.04), and HOMA-IR (*r* = 0.48, *P* = 0.05). PEA was correlated with fasting insulin (*r* = 0.49, *P* = 0.04). 2-AG had a negative correlation with fasting glucose (*r* = −0.59, *P* = 0.04).* Conclusion*. Gender differences exist in circulating ECs in obese subjects. Females show changes in AEA and PEA after bariatric surgery. Specific correlations exist between different ECs and markers of obesity and insulin and glucose homeostasis.

## 1. Introduction

Recently, considerable interest has developed in the role that the endocannabinoid system (ECS) plays in obesity, impaired glucose homeostasis, and type 2 diabetes mellitus (T2DM) [1]. The ECS comprises the endocannabinoids (ECs), the enzymes involved in their synthesis and degradation, and the cannabinoid (CB) receptors which are activated by ECs [2]. ECs are bioactive lipid mediators that are synthesised and released on demand [3]. The most comprehensively studied ECs are the fatty acid amides, N-arachidonoyl ethanolamine (AEA), N-palmitoylethanolamine (PEA), N-oleoylethanolamine (OEA), and related N-acylethanolamine (NAE) derivatives together with esters of arachidonic acid including 2-arachidonoylglycerol (2-AG) [4–7]. The ECS is critically important in the control and regulation of body weight both centrally and peripherally. For example, activation of CB_1_ by AEA and 2-AG is associated with increased food intake, weight gain, and obesity, whilst OEA and PEA are associated with the suppression of these effects. Debate exists on whether PEA and OEA can be considered as endocannabinoid ligands as they have no activity at the classic cannabinoid receptors (CB_1_ and CB_2_) [8]. Increased levels of ECs have been observed in the circulation and visceral fat stores in obese subjects [9]; furthermore, adipocytes may synthesise ECs* de novo *[10]. Obese humans have elevated circulating levels of AEA and 2-AG compared to nonobese controls of both genders [11–13]. A previous study has shown higher circulating levels of 2-AG in insulin resistant obese postmenopausal women [14] and in obese younger females [15]. Overweight subjects with T2DM and hyperglycaemia also have higher circulating ECs compared to age- and body mass index- (BMI-) matched normoglycaemic subjects [10, 16].

Obesity is a major risk factor for insulin resistance and T2DM. Activation of the peripheral ECS has been observed in human obesity [11], resulting in adipogenesis, lipogenesis, hepatic steatosis, and increased insulin resistance [17, 18]. AEA is metabolised by fatty acid amide hydrolase (FAAH). Conflict exists within the literature with respect to FAAH activity in obesity. Studies have described reduced expression of FAAH in obese insulin resistant subjects [19]; other studies have observed a positive correlation between FAAH activity and BMI in a healthy population [20], but not in a bariatric sample of subjects [2]. The former observation of reduced FAAH in obese insulin resistant subjects together with the observation that increased adiposity or BMI may be associated with increased plasma ECs might result in a further incremental increase in ECs [12, 13, 21]. Sipe et al. demonstrated that circulating ECs predict obesity risk and suggested targeting ECs as a novel strategy for treating obesity [22]. Furthermore, CB_1_ receptor blockade ameliorates disturbances in glucose and insulin metabolism in mice fed a high-fat diet [23]. CB_1_ and CB_2_ receptor antagonists such as rimonabant have been studied within phase 3 clinical trials [24].

Bariatric surgery is an effective treatment for morbid obesity and is associated with reduced calorie intake, significant weight loss, and resolution of T2DM [25]. Little information is available on the effects of extreme weight loss, such as that associated with bariatric surgery in relation to the ECS. Of interest, a modest weight loss of 5% by diet or medication (sibutramine) did not influence circulating EC levels in postmenopausal women or obese subjects, respectively [11, 26], whereas a 10% weight reduction in abdominally obese men was associated with a reduction in 2-AG levels [27]. Downregulation of AEA and 2-AG following Roux-en-Y gastric bypass has been demonstrated in a rat model [28]. Furthermore, the effect of surgical gastric bypass-induced weight loss on changes in ECs on coronary circulatory dysfunction in morbidly obese individuals has been studied [29]. The aim of our current study was to examine gender differences in circulating ECs and changes in circulating ECs in relation to bariatric surgery (as an extreme weight loss intervention). ECs were measured preoperatively and 6 months postoperatively. We also studied the association between circulating ECs and markers of obesity and insulin and glucose homeostasis pre- and postoperatively.

## 2. Material and Methods

### 2.1. Study Participants

Approval for the study was obtained from the Local Research Ethics Committee (South West Wales; LREC reference 06/WMW02/7) and the Joint Scientific Research Committee at Swansea University and ABM University Health Board. Participants were identified and recruited from patients undergoing a planned bariatric surgical procedure at the Welsh Institute of Metabolic and Obesity Surgery (WIMOS) at Morriston Hospital, ABM University Health Board, Swansea, Wales, UK. Informed consent was obtained by trained personnel. Entry criteria at the outset of the study included both sexes, age > 18 years, BMI > 40 Kg/m^2^, and being physically fit for surgery. All subjects had either previously diagnosed T2DM, impaired glucose regulation, or T2DM diagnosed during an oral 75 g glucose tolerance test (OGTT) at the start of the study. Participants with impaired glucose regulation were those with either impaired fasting glycaemia (IFG, 5.6–6.9 mmol/L) or impaired glucose tolerance (IGT, 2-hour glucose: 7.8–11.0 mmol/L) [30]. Participants with preexisting diabetes treated with diet, oral agents, GLP-1 analogues, or insulin were included. Participants with any acute concurrent illness were excluded. At the time of recruitment no participant volunteered a history of current or past cannabis use. We did not measure plasma cannabinoid levels.

### 2.2. Study Design

Participants were recruited prospectively from the bariatric surgical clinic and were not blindly allocated to a surgical treatment option. As per local guidance (within WIMOS) at the time, those with a BMI > 50 Kg/m^2^ were routinely offered a biliopancreatic diversion (BPD), whereas those with a BMI below this were offered laparoscopic sleeve gastrectomy (LSG). LSG was performed as a standard procedure, that is, sleeve fashioned around a 32F bougie taken from 5 cm proximal to the pylorus and up to the left crus. BPD involved a distal gastrectomy (as described by Scopinaro et al. [31]) and a 50 cm common channel. All participants were recruited 1 month preoperatively and followed up postoperatively at 6 months. Patients underwent a standardised 75 g oral glucose tolerance test (OGTT) (122 mLs of Polycal 61.9 g/100 mL of glucose, Nutricia Clinical Care, Trowbridge, UK) pre- and postoperatively. There was no standardised meal prescribed for the night before and subjects were asked to fast from the midnight before the test with diabetes related medication omitted.

### 2.3. Baseline Clinical and Biochemical Information

At the time of the preoperative and postoperative OGTT the following clinical information was ascertained: age, gender, past medical history, treatment, and duration of diabetes. Baseline clinical measurements consisted of weight, height, BMI, waist circumference, and systolic and diastolic blood pressure. Baseline biochemical measurements (total cholesterol, low density lipoprotein-cholesterol (LDL-C), high density lipoprotein-cholesterol (HDL-C), and triglycerides) were performed within the local hospital accredited laboratory. Fasting and 2-hour glucose, fasting lipids (Roche Modular P800 Analyser), and fasting insulin and C-peptide (Roche E170 Modular Analyser) were also measured in the local hospital laboratory. All samples were collected on ice, centrifuged and separated within one hour of collection, and subsequently stored at −80°C until analysis.

### 2.4. Measurements of Insulin Sensitivity

The Homeostasis Model Assessment (HOMA) was used to estimate steady state beta cell function (%B) and insulin sensitivity (%S) preoperatively and postoperatively. These were calculated using the Oxford University online calculator (http://www.dtu.ox.ac.uk/homacalculator/, accessed May 7, 2013) [32]. HOMA-IR is the reciprocal of HOMA %S. HOMA %S represent values of 100% in normal young adults when using currently available assays for insulin, specific insulin, or C-peptide. The accuracy of these measures has been validated and has been shown to correlate with clamp-derived indices of insulin sensitivity and secretion [33]. The fasting C-peptide to insulin ratio was calculated as an index of hepatic insulin clearance [34].

### 2.5. Measurement of Plasma Endocannabinoid Levels

An LC-ESI^+^-MS/MS method was used for analysis of ECs in human plasma samples based on a previously published method [35]. Fasting plasma samples were obtained preoperatively and postoperatively. Plasma was separated immediately and stored at −80°C immediately. Internal standards of 100 *μ*L of 2-AG-d8 (10 *μ*M) and 15 *μ*L of AEA-d8 (28 *μ*M) were added to aliquots of each plasma (0.4 mL) sample or blank sample (0.4 mL water) or EC standards and vortexed briefly. Ethyl acetate : hexane (9 : 1 v/v) was added to each sample, vortex-mixed (10 min), and centrifuged (13000 rpm, 10 min, 4°C). The procedure was repeated and the supernatants pooled and evaporated using a vacuum centrifugal evaporator. Prior to analysis, each sample extract was reconstituted in 100 *μ*L of acetonitrile. The injection volume was 10 *μ*L. EC standards (AEA, 2-AG, OEA, PEA, and internal standards 2-AG-d8, AEA-d8) were purchased from Axxora Laboratory services, Bingham, Nottingham, UK. The HPLC system used was a Shimadzu SCL 10Avp (Shimadzu, Columbia, MD, USA) coupled to a triple quadrupole ion-trap 4000 QTRAP mass-spectrometer (ABI, UK) equipped with Turbo Spray ionisation interface. Analytes were separated chromatographically on a Waters Symmetry C18 column (internal diameter 100 × 2.1 mm, particle size 3.5 *μ*m) with a mobile phase flow rate of 0.3 mL/minute. Multiple-reaction monitoring of individual compounds using specific precursor and product mass-to-charge (*m/z*) ratios allowed simultaneous measurement of AEA, 2-AG, OEA, and PEA.

### 2.6. Statistical Methods

Statistical analysis was performed using SPSS. Continuous variables that did not have a normal distribution (AEA, OEA, PEA, and 2-AG) underwent log transformation to normalise the data for analysis and are described with the geometric mean and approximate standard deviation. For continuous variables, differences in means were compared pre- and postoperatively using a paired sample *t*-test. Differences between males and females were compared by ANOVA. We also examined the correlation (Spearman's correlation) between BMI, weight, waist circumference, fasting plasma glucose, 2-hour plasma glucose, glycated haemoglobin (HbA_1c_), systolic and diastolic blood pressure, fasting insulin, and markers of insulin resistance with plasma ECs pre- and postoperatively. In all cases a *P* < 0.05 was considered statistically significant.

As our aim was to examine the effects of bariatric surgery as an extreme weight loss intervention, we chose from the outset to examine the surgical groups together rather than separate. Our aim was therefore not to compare the effects of LSG and BPD. This would have also reduced the numbers to 10 within each group. As there are no previous studies performed in this group of subjects, a baseline power calculation was not performed. We also chose from the outset to examine for gender differences in circulating ECs.

## 3. Results

### 3.1. Participant Characteristics

Samples from twenty participants were analysed. Of these, 10 underwent LSG and 10 underwent BPD. There were 10 females and 10 males with a mean age of 49.8 ± 7 years. The baseline clinical characteristics pre- and postoperatively are shown in Table 1.

### 3.2. Weight, Blood Pressure, Lipids, and Glucose Control Pre- and Postoperatively

As shown in Table 1 and in line with previous data, there was a significant mean weight reduction of 35.4 kg at 6 months. This was associated with significant reductions in waist circumference and BMI at 6 months. No statistically significant changes were observed in systolic and diastolic blood pressure, total cholesterol, LDL-C, and triglyceride levels postoperatively.

### 3.3. Effects of Bariatric Surgery on Glucose and Markers of Insulin Resistance

As shown in Table 1, significant changes were observed in 2-hour plasma glucose and HbA_1c_ at 6 months. Following surgery, 20% of the subjects had T2DM compared to 65% prior to surgery. There was a significant reduction in the fasting insulin and an increase in hepatic insulin clearance at 6 months postoperatively. HOMA %S was significantly increased at 6 months. There was no significant change in fasting C-peptide following surgery.

### 3.4. Effects of Bariatric Surgery on Circulating Plasma Endocannabinoid Levels

In the sample as a whole, there were no significant changes in circulating levels of AEA, OEA, PEA, and 2-AG postoperatively (Table 1). We observed significant differences in circulating levels of AEA, OEA, and PEA between males and females preoperatively (Table 2). Furthermore, in the females, significant reductions were observed in postoperative circulating AEA and PEA compared to preoperative circulating AEA and PEA (Table 2). Of interest, the postoperative levels in the females were comparable to the levels in the males pre- and postoperatively. No gender differences in circulating ECs were observed postoperatively.

### 3.5. Temporal Correlations between BMI, Glycaemic Control, Insulin Resistance, and Endocannabinoids

As described within the methods, we chose from the outset to examine the correlation between markers of obesity and insulin and glucose homeostasis and ECs preoperatively and postoperatively. The results are shown in Table 3. For AEA, preoperatively, there were significant positive correlations with 2-hour plasma glucose (*r* = 0.55, *P* = 0.01), HOMA-IR (*r* = 0.61, *P* = 0.009), and a negative correlation with HOMA %S (*r* = −0.71, *P* = 0.002); these were no longer present postoperatively. Preoperatively, OEA had a significant correlation with weight (*r* = 0.49, *P* = 0.03), waist circumference (*r* = 0.52, *P* = 0.02), fasting insulin (*r* = 0.49, *P* = 0.04), and HOMA-IR (*r* = 0.48, *P* = 0.05). With respect to PEA, preoperatively, there was a positive correlation with fasting insulin (*r* = 0.49, *P* = 0.04) and LDL-C (*r* = 0.44, *P* = 0.04) and 2-AG had a negative correlation with fasting plasma glucose (*r* = −0.59, *P* = 0.04).

With respect to the circulating ECs, preoperatively, AEA had significant correlations with OEA and PEA (*r* = 0.52, *P* = 0.02 and *r* = 0.71, *P* < 0.001) and 6 months postoperatively (*r* = 0.60, *P* = 0.005 and *r* = 0.61, *P* = 0.005, resp.).

## 4. Discussion

In line with previous studies, we observed significant improvements in T2DM following bariatric surgery [36–38]. Using the American Diabetes Association criteria for the diagnosis of diabetes based on plasma glucose, we observed that 80% of participants had normal glucose levels during the postoperative OGTT. Within the current study group, we observed significant improvements postoperatively in glycaemic control, insulin sensitivity (HOMA %S), and hepatic insulin clearance.

Our aim was to examine changes in circulating ECs in relation to bariatric surgery (as a weight loss intervention), differences by gender, and the association between plasma ECs and markers of insulin resistance before and after bariatric surgery. In the whole group, prior to gender stratification, we observed no changes in circulating AEA, OEA, PEA, and 2-AG before and after bariatric surgery. This is in line with previous studies where a modest weight loss of 5% by diet or medication (sibutramine) did not influence circulating ECs in postmenopausal women or obese subjects, respectively [11, 26], but contradicts the effect of 10% weight loss on 2-AG and AEA levels reported for obese men [27] and an increase in PEA in postmenopausal women [14] with a mean BMI of 32.9 Kg/m^2^. This may be due to the extremely high BMI both pre- and postoperatively despite surgical induced weight loss and may be unique to our study sample. We observed significant gender differences preoperatively which is in line with a previous study which described gender differences in circulating levels of AEA, OEA, and PEA [14]. Postoperatively, we observed significant reductions in circulating AEA and PEA in females but not males. Of note, there was no significant difference in weight, BMI, and waist circumference between females and males preoperatively or postoperatively. Furthermore, no significant differences were observed between gender (females versus males) for changes postoperatively in weight (38 ± 18 versus 33 ± 24 kg, *P* = 0.58), BMI (14.0 ± 7.5 versus 9.6 ± 7.0 kg/m^2^, *P* = 0.20), and waist circumference (21.3 ± 6.8 versus 19.2 ± 6.8 cm, *P* = 0.52). No gender differences were seen for postoperative changes in fasting glucose, 2-hour glucose, fasting insulin, HbA_1c_, and HOMA measurements. Therefore, the reason for this gender difference is unclear but is likely to be related to an interaction between sex hormones and the ECS. This is supported by previous literature [39, 40] which supports a role for female sex hormones in modulating EC activity. We would therefore suggest that, for further studies examining circulating ECs, gender stratification is essential. A limitation of our current study was that we did not measure sex hormones nor did we document menstrual history, but none of the female participants had reported menopausal symptoms.

There is paucity in the published literature examining the associations between circulating ECs and markers of fat mass and glucose and insulin homeostasis. We observed distinct associations between specific ECs and these markers. Previous studies have shown that weight and waist circumference are accepted markers of insulin resistance [41] and that circulating EC levels are associated with insulin resistance [10, 16, 42]. Preoperatively, AEA had significant correlations with 2-hour plasma glucose and markers of insulin resistance. OEA was correlated with weight, waist circumference, and insulin resistance. PEA had a positive correlation insulin and 2-AG with fasting glucose. Therefore, the different ECs appear to be associated with different markers of fat mass and glucose and insulin homeostasis. However, AEA was correlated with OEA and PEA. Therefore, there appear to be complex and specific biochemical-clinical relationships. These findings have not been described previously and warrant further investigation. None of the above correlations were observed postoperatively. One possible explanation for this may be related to a change in diet following bariatric surgery. A recent study has shown that obesity and high fat dietary intake influences CB_1_ receptor expression in skeletal muscle and FAAH gene expression in subcutaneous adipose tissue [43]. These effects were not observed in lean and low fat diets. Another explanation is that genetic variation within CB_1_ receptor may influence metabolic function in response to changes in diet or medication [44, 45]. Another possible explanation may be related to changes in other satiety hormones which influence the ECS. These may include ghrelin and leptin, both of which are influenced by bariatric surgery [46, 47] and other means of weight reduction [48]. This is an area which warrants further investigation.

Therapeutic interventions which target the ECS through the blockade of CB_1_ receptors have been extensively studied and have shown benefits in glucose metabolism and insulin resistance [21, 23, 49]. To the best of our knowledge, this current study is the only one that has examined the relationship of ECs following extreme weight loss resulting from bariatric surgery. Further work is required to explore these findings to establish whether circulating ECs may have a causal association with obesity and impaired glucose and insulin homeostasis or whether they are merely a marker of increased fat mass and associated metabolic dysregulation. The gender differences relating to the ECs also require more detailed examination. The current study adds to the available literature examining the role of ECs in obesity and glucose and insulin homeostasis. A limitation of our current study is that this comprised a small number of 20 subjects. In view of this number, we combined the two types of surgeries which we acknowledge involve dissimilar mechanisms to induce weight loss. The study was explorative in nature and the data will add to the potential for a more robust larger powered study within this interesting field of investigation. Furthermore, future studies should examine the different bariatric procedures in relation to circulating ECs.

## Figures and Tables

**Table 1:** Pre- and postoperative clinical and biochemical measurements.

Measurement	Preoperative (*n* = 20)	Postoperative (*n* = 20)	*P*
Weight (kg)	160.2 (44.2)	124.8 (29.4)	<0.001
BMI (kg/m^2^)	57.3 (14.1)	45.4 (10.1)	<0.001
Waist (cm)	143.7 (21.5)	123.4 (20.1)	<0.001
Systolic BP (mmHg)	133 (18)	128 (16)	0.19
Diastolic BP (mmHg)	79 (9)	75 (10)	0.06
Cholesterol (mmol/L)	4.2 (0.9)	3.9 (1.1)	0.26
LDL-C (mmol/L)	2.2 (0.8)	2.1 (0.9)	0.73
HDL-C (mmol/L)	1.2 (0.3)	1.1 (0.3)	0.08
Triglyceride (mmol/L)	1.7 (0.9)	1.4 (0.4)	0.18
HbA_1c_ (%)	7.2 (1.5)	6.1 (1.3)	0.02
HbA_1c_ (mmol/L)	55 (43.1)	43 (37.3)	0.02
Fasting glucose (mmol/L)	7.8 (3.3)	6.0 (3.1)	0.13
2-hour glucose (mmol/L)	12.7 (4.9)	8.3 (5.9)	0.01
Fasting insulin (IU/mL)	27.9 (16.2)	11.4 (6.7)	0.002
Fasting C-peptide (ng/mL)	3.6 (1.5)	2.9 (1.5)	0.10
HOMA %S	305.8 (208.3)	617.5 (492.5)	0.03
HOMA- IR	0.4667 (0.3016)	0.3600 (0.4154)	0.32
C-pep: Insulin ratio	0.1744 (0.0996)	0.2741 (0.1246)	0.001
AEA (pmol/mL)^*^	0.21 (0.04)	0.19 (0.04)	0.33
OEA (pmol/mL)^*^	1.05 (0.13)	0.93 (0.18)	0.25
PEA (pmol/mL)^*^	0.83 (0.09)	0.74 (0.09)	0.10
2-AG (pmol/mL)^*^	5.0 (1.55)	4.2 (1.40)	0.14

^*^Geometric mean and approximate standard deviation shown for log transformed data.

**Table 2:** Pre- and postoperative gender differences in ECs.

Measurement	Preoperative			Postoperative		
	Females	Males	*P*	Females	Males	*P*
AEA (pmol/mL)^*^	0.297 (0.042)	0.147 (0.021)	<0.001	0.209 (0.049)^†^	0.165 (0.033)	0.30
OEA (pmol/mL)^*^	1.263 (0.125)	0.869 (0.085)	0.002	0.947 (0.232)	0.912 (0.127)	0.86
PEA (pmol/mL)^*^	0.964 (0.096)	0.720 (0.054)	0.005	0.759 (0.110)^††^	0.713 (0.084)	0.67
2-AG (pmol/mL)^*^	5.163 (1.831)	4.019 (2.184)	0.43	5.156 (2.086)	3.249 (0.840)	0.51

^*^Geometric and approx. SD shown. Log transformed.

^†^ *P* = 0.02, preoperative versus 6 months postoperatively.

^††^ *P* = 0.007, preoperative versus 6 months postoperatively.

**Table 3:** Correlations between circulating ECs and markers of obesity and insulin and glucose homeostasis pre- and postoperatively.

Variable	AEA	OEA	PEA	2-AG
BMI				
0 months	0.17 (0.68)	0.42 (0.06)	0.30 (0.19)	0.07 (0.45)
6 months	0.24 (0.32)	0.17 (0.50)	−0.21 (0.93)	−0.16 (0.53)
Weight				
0 months	0.10 (0.68)	**0.49 (0.03)**	0.36 (0.12)	0.20 (0.45)
6 months	0.01 (0.96)	0.01 (0.97)	−0.23 (0.35)	−0.16 (0.53)
Waist				
0 months	0.10 (0.68)	**0.52 (0.02)**	0.40 (0.08)	0.26 (0.33)
6 months	−0.13 (0.62)	−0.04 (0.86)	−0.28 (0.25)	0.11 (0.70)
Fasting glucose				
0 months	0.15 (0.55)	0.05 (0.84)	0.09 (0.71)	**−0.59 (0.04)**
6 months	0.54 (0.05)	0.11 (0.62)	0.18 (0.46)	−0.09 (0.75)
2-hour glucose				
0 months	**0.55 (0.01)**	0.11 (0.65)	0.17 (0.47)	0.003 (1.00)
6 months	−0.02 (0.94)	−0.24 (0.33)	−0.13 (0.60)	−0.42 (0.10)
HbA_1c_				
0 months	0.44 (0.06)	0.22 (0.37)	0.20 (0.40)	−0.12 (0.66)
6 months	0.09 (0.71)	−0.13 (0.59)	−0.00 (0.97)	−0.35 (0.17)
Fasting insulin				
1 month	0.28 (0.26)	**0.49 (0.04)**	**0.49 (0.04)**	0.18 (0.54)
6 months	−0.03 (0.89)	0.06 (0.81)	0.08 (0.76)	−0.19 (0.49)
HOMA S%				
1 month	**−0.71 (0.002)**	−0.34 (0.18)	−0.47 (0.06)	0.16 (0.57)
6 months	−0.35 (0.16)	−0.25 (0.33)	−0.33 (0.18)	−0.21 (0.44)
HOMA- IR				
0 months	**0.61 (0.009)**	**0.48 (0.05)**	**0.53 (0.03)**	0.27 (0.32)
6 months	0.40 (0.1)	0.28 (0.27)	0.25 (0.31)	0.38 (0.15)
Systolic BP				
0 months	0.40 (0.09)	0.11 (0.66)	0.25 (0.30)	−0.01 (1.0)
6 month	−0.13 (0.61)	−0.27 (0.26)	−0.06 (0.81)	−0.07 (0.78)
Diastolic BP				
0 months	**0.48 (0.04)**	0.01 (0.96)	0.14 (0.56)	**−0.52 (0.04)**
6 months	−0.06 (0.83)	−0.08 (0.76)	0.08 (0.74)	−0.23 (0.38)
LDL-C				
0 months	0.15 (0.54)	0.15 (0.52)	**0.44 (0.04)**	−0.02 (0.94)
6 months	−0.27 (0.27)	−0.26 (0.28)	−0.04 (0.87)	0.13 (0.62)
HDL-C				
0 months	−0.21 (0.37)	−0.12 (0.53)	−0.31 (0.18)	**−0.73 (0.001)**
6 months	−0.32 (0.18)	0.18 (0.45)	0.28 (0.25)	0.03 (0.91)
2-AG				
0 months	0.08 (0.76)			
6 months	−0.01 (0.98)			
OEA				
0 months	**0.52 (0.02)**			
6 months	**0.60 (0.005)**			
PEA				
0 months	**0.71 (<0.001)**			
6 months	**0.61 (0.005)**			

The *r* values are shown with the *P* values in brackets. Significant correlations are shown in bold.
